# Supplementary material for: Comparative genomics provides new insights into the diversity, physiology, and sexuality of the only industrially exploited tremellomycete: Phaffia rhodozyma
Source: BMC Genomics. 2016 Nov 9;17:901. doi: 10.1186/s12864-016-3244-7 (PMC5103461; doi:10.1186/s12864-016-3244-7)
Supplement: Additional file 6: — List of orphan genes with links to PFAM (related to Additional file 1: Table S1). (ZIP 1428 kb) [file 12864_2016_3244_MOESM6_ESM.zip › PFAM_UNIQUE/G02129_P.html]

#                                                                            --- full sequence --- -------------- this domain -------------   hmm coord   ali coord   env coord
  
# target name        accession   tlen query name           accession   qlen   E-value  score  bias   #  of  c-Evalue  i-Evalue  score  bias  from    to  from    to  from    to  acc description of target
  
#------------------- ---------- ----- -------------------- ---------- ----- --------- ------ ----- --- --- --------- --------- ------ ----- ----- ----- ----- ----- ----- ----- ---- ---------------------
  
G02129\_P             -            585 AAA\_13               PF13166.1    712      0.52    7.4   4.3   1   1     0.006      0.94    6.5   4.3   273   358   195   278   183   349 0.79 - AAA domain  
G02129\_P             -            585 ARGLU                PF15346.1    149     0.063   11.7   1.2   1   1   0.00032      0.12   10.8   1.2    40   132   188   279   161   293 0.75 - Arginine and glutamate-rich 1  
G02129\_P             -            585 CENP-F\_leu\_zip       PF10473.4    140       3.3    6.3   9.7   1   2     0.046       8.9    4.8   5.5    80   116   242   278   201   310 0.55 - Leucine-rich repeats of kinetochore protein Cenp-F/LEK1  
G02129\_P             -            585 CENP-F\_leu\_zip       PF10473.4    140       3.3    6.3   9.7   2   2      0.19        36    2.9   0.1    21    67   498   550   492   583 0.80 - Leucine-rich repeats of kinetochore protein Cenp-F/LEK1  
G02129\_P             -            585 CENP-Q               PF13094.1    160       6.3    5.5   9.3   1   2       2.2   6.9e+02   -1.2   0.0   110   145   191   225   148   235 0.71 - CENP-Q, a CENPA-CAD centromere complex subunit  
G02129\_P             -            585 CENP-Q               PF13094.1    160       6.3    5.5   9.3   2   2     0.026       8.1    5.1   7.1    48    97   242   291   233   332 0.83 - CENP-Q, a CENPA-CAD centromere complex subunit  
G02129\_P             -            585 DUF3584              PF12128.3   1201      0.29    7.1   5.9   1   1    0.0032      0.44    6.5   5.9   649   763   205   320   187   348 0.74 - Protein of unknown function (DUF3584)  
G02129\_P             -            585 DUF4600              PF15372.1    130       1.3    7.9   5.4   1   1    0.0012       3.7    6.5   5.4    13   104   193   285   184   332 0.51 - Domain of unknown function (DUF4600)  
G02129\_P             -            585 Filament             PF00038.16   312      0.29    9.2   4.9   1   1    0.0046      0.74    7.9   4.9   180   260   187   279   183   300 0.77 - Intermediate filament protein  
G02129\_P             -            585 GAS                  PF13851.1    201      0.67    7.8   6.0   1   2    0.0023      0.43    8.4   3.9    51   126   238   312   202   329 0.84 - Growth-arrest specific micro-tubule binding  
G02129\_P             -            585 GAS                  PF13851.1    201      0.67    7.8   6.0   2   2       9.7   1.9e+03   -3.5   0.0   172   190   529   547   497   550 0.72 - Growth-arrest specific micro-tubule binding  
G02129\_P             -            585 Mnd1                 PF03962.10   188       4.3    5.6   6.3   1   1     0.032       9.4    4.5   5.8    62   147   242   330   209   337 0.81 - Mnd1 family  
G02129\_P             -            585 Rep-A\_N              PF04057.7    101     0.032   12.7   0.1   1   1   4.3e-05     0.086   11.3   0.1    25    65   214   257   205   265 0.85 - Replication factor-A protein 1, N-terminal domain  
G02129\_P             -            585 Sec2p                PF06428.6    101     0.096   11.2   1.8   1   1   0.00033      0.28    9.7   1.8    39    90   237   289   234   302 0.84 - GDP/GTP exchange factor Sec2p  
G02129\_P             -            585 Seryl\_tRNA\_N         PF02403.17   108       9.3    5.0   9.6   1   2     0.011       3.4    6.4   4.9    40    98   210   273   188   286 0.76 - Seryl-tRNA synthetase N-terminal domain  
G02129\_P             -            585 Seryl\_tRNA\_N         PF02403.17   108       9.3    5.0   9.6   2   2       1.5   4.5e+02   -0.4   0.1    48    85   533   571   513   584 0.61 - Seryl-tRNA synthetase N-terminal domain  
G02129\_P             -            585 SF-assemblin         PF06705.6    247       6.4    4.5   8.9   1   2    0.0062       6.2    4.5   5.6    33   113   226   310   215   356 0.83 - SF-assemblin/beta giardin  
G02129\_P             -            585 SF-assemblin         PF06705.6    247       6.4    4.5   8.9   2   2      0.16   1.6e+02   -0.1   0.0   104   149   499   544   492   554 0.82 - SF-assemblin/beta giardin  
G02129\_P             -            585 Spc24                PF08286.6    118      0.35    9.2   4.4   1   1    0.0014      0.83    8.0   4.4     5    60   227   279   224   286 0.81 - Spc24 subunit of Ndc80  
G02129\_P             -            585 TBPIP                PF07106.8    169      0.17   10.1   4.0   1   1    0.0012      0.44    8.7   4.0    58   159   181   292   179   306 0.68 - Tat binding protein 1(TBP-1)-interacting protein (TBPIP)  
G02129\_P             -            585 TipAS                PF07739.8    118      0.16   11.0   3.2   1   2   0.00014      0.27   10.2   1.9    17    72   202   257   182   294 0.80 - TipAS antibiotic-recognition domain  
G02129\_P             -            585 TipAS                PF07739.8    118      0.16   11.0   3.2   2   2       2.9   5.8e+03   -3.7   0.0   106   116   506   516   504   517 0.85 - TipAS antibiotic-recognition domain  
G02129\_P             -            585 Tropomyosin          PF00261.15   237       1.7    6.3   4.8   1   1     0.014       3.4    5.4   4.8     6    63   218   276   213   289 0.55 - Tropomyosin  
G02129\_P             -            585 Tropomyosin\_1        PF12718.2    143      0.88    8.1   5.3   1   2    0.0014      0.47    9.0   2.6    44    88   244   286   210   329 0.60 - Tropomyosin like  
G02129\_P             -            585 Tropomyosin\_1        PF12718.2    143      0.88    8.1   5.3   2   2       3.2   1.1e+03   -1.9   0.0    43    90   495   545   491   581 0.55 - Tropomyosin like  
G02129\_P             -            585 TSKS                 PF15358.1    558     0.078    9.8   0.8   1   1     7e-05      0.14    8.9   0.8   355   413   233   291   209   309 0.86 - Testis-specific serine kinase substrate  
G02129\_P             -            585 V\_ATPase\_I           PF01496.14   759      0.83    6.1   4.1   1   1    0.0039       1.2    5.6   4.1    50   134   213   302   192   358 0.72 - V-type ATPase 116kDa subunit family  
G02129\_P             -            585 YkyA                 PF10368.4    204      0.59    8.1   3.8   1   1    0.0014       1.2    7.1   3.8    21   126   238   348   234   361 0.79 - Putative cell-wall binding lipoprotein
